# Prosthetic liner wear in total hip replacement: a longitudinal 13-year study with computed tomography

**DOI:** 10.1007/s00256-018-2878-8

**Published:** 2018-01-23

**Authors:** Lars Weidenhielm, Henrik Olivecrona, Gerald Q. Maguire, Marilyn E. Noz

**Affiliations:** 1 Department of Molecular Medicine and Surgery, Karolinska Institute, Stockholm, Sweden; 2 School of Information and Communication Technology, KTH Royal Institute of Technology, Stockholm, Sweden; 3 Department of Radiology, New York University, New York, NY USA

**Keywords:** Hip replacement, Computed tomography, Prosthetic liner wear

## Abstract

This case report follows a woman who had a total hip replacement in 1992 when she was 45 years old. Six serial computed tomography (CT) examinations over a period of 13 years provided information that allowed her revision surgery to be limited to liner replacement as opposed to replacement of the entire prosthesis. Additionally, they provided data that ruled out the presence of osteolysis and indeed none was found at surgery. In 2004, when the first CT was performed, the 3D distance the femoral head had penetrated into the cup was determined to be 2.6 mm. By 2017, femoral head penetration had progressed to 5.0 mm. The extracted liner showed wear at the thinnest part to be 5.5 mm, as measured with a micrometer. The use of modern CT techniques can identify problems, while still correctable without major surgery. Furthermore, the ability of CT to assess the direction of wear revealed that the liner wear changed from the cranial to dorsal direction.

## Introduction

Millions of people worldwide have had total hip replacements and especially in younger patients like ours, predominately uncemented prosthetic replacements were used. When our patient was operated on, uncemented cups were associated with many problems such as inferior quality polyethylene, thin liners, and bad locking mechanisms. This often resulted in high wear rate causing periacetabular osteolysis and aseptic cup loosening [1]. As patients are living longer and are more active, there is an increasing probability that the patient will need a revision due to wear and osteolysis. Wear and osteolysis are silent diseases and severe bone loss can occur before the patient’s hip symptoms bring him/her to the doctor. This might result in an extensive revision [2]. However, there is no consensus on how we should follow these patients. Plain radiographs are not sufficiently sensitive for detection of and description of periacetabular osteolysis [1, 3, 4]. Computed tomography (CT) is a far more accurate method, but high radiation levels were a major concern [1, 5]. Today, prosthetic liner wear and osteolysis can be assessed with clinically acceptable accuracy at a CT radiation level on par with conventional plain radiographic examination [6]. Furthermore, CT offers a three-dimensional assessment of the liner wear in contrast to conventional radiographic examination where two-dimensional measurements may underestimate polyethylene wear [7, 8].

This study presents a case of a woman who, in 1992, at the age of 45, had a total hip arthroplasty (THA) and who was subsequently followed with CT. We show the progression of liner wear as seen between years 2004 to 2017. In this patient, osteolysis was suspected on planar X-ray, but was not seen on CT, and was not present at the operation. This report demonstrates that, with the use of modern CT techniques, especially in patients who had THA when young, problems can be identified while still correctable without major surgery.

## Case report

On January 14, 1992, a 45-year-old woman with osteoarthritis in her left hip was operated on in the Hospital for Special Surgery, NY. An uncemented hip replacement was used. According to the original operative record, the prosthesis was a 58-mm Harris-Galante II acetabular cup (Zimmer, Warsaw, IN, USA) fixed with two screws. An elevated polyethylene liner with a thickness of 10 mm and an Osteonics femoral stem No. 7 (Stryker Howmedica Osteonics, Mahwah, NJ, USA) with a 28-mm plus 10 prosthetic metal femoral head and neck completed the prosthesis. The postoperative course was uneventful and she had a good clinical result with only slight pain after long walks. Seven years after the operation, her hip prosthesis dislocated during severe rotation when she was practicing tai-chi. It was managed by closed reduction and radiographic examination indicated slight wear of the polyethylene in the cup. Osteolysis around the cup was also suspected on conventional radiographic examination. A discussion about revision of the cup due to wear and osteolysis started and resulted in a referral to our department 17 years ago. At that time, we had started to assess wear and osteolysis around the cup with CT and she had her first examination in October 2004. We found no osteolysis around the cup. Liner wear defined as maximal femoral head penetration into the polyethylene liner was 2.6 mm measured on the CT scan. This meant that there was 7.4 mm polyethylene left in the most worn part of the cup. Since her hip was pain free and her hip function was excellent, we decided to not revise the hip prosthesis but instead follow her with CT examinations of the operated hip. In 2005, the polyethylene wear had progressed to 3 mm. In 2007, the wear was 3.2 mm and in 2011 it was 3.9 mm. Her clinical function was excellent on all these follow-up examinations. In 2011, she was operated on for a leiomyosarcoma in her ventricle. She had no metastasis and did well on the follow-up examinations at the oncology center where she was treated but she forgot about her hip examinations. In 2016, she returned to our department. She had slight pain in her left hip and she had noted a swelling in her groin. On ultrasound examination, a cyst was found in her left groin. A CT examination of her left hip showed that wear had progressed to 4.5 mm, no osteolysis around the cup, but did not show the cyst. The cyst was suspected to be due to wear particles and due to the cyst and her hip pain we decided to revise her hip prosthesis.

**Fig. 1:**
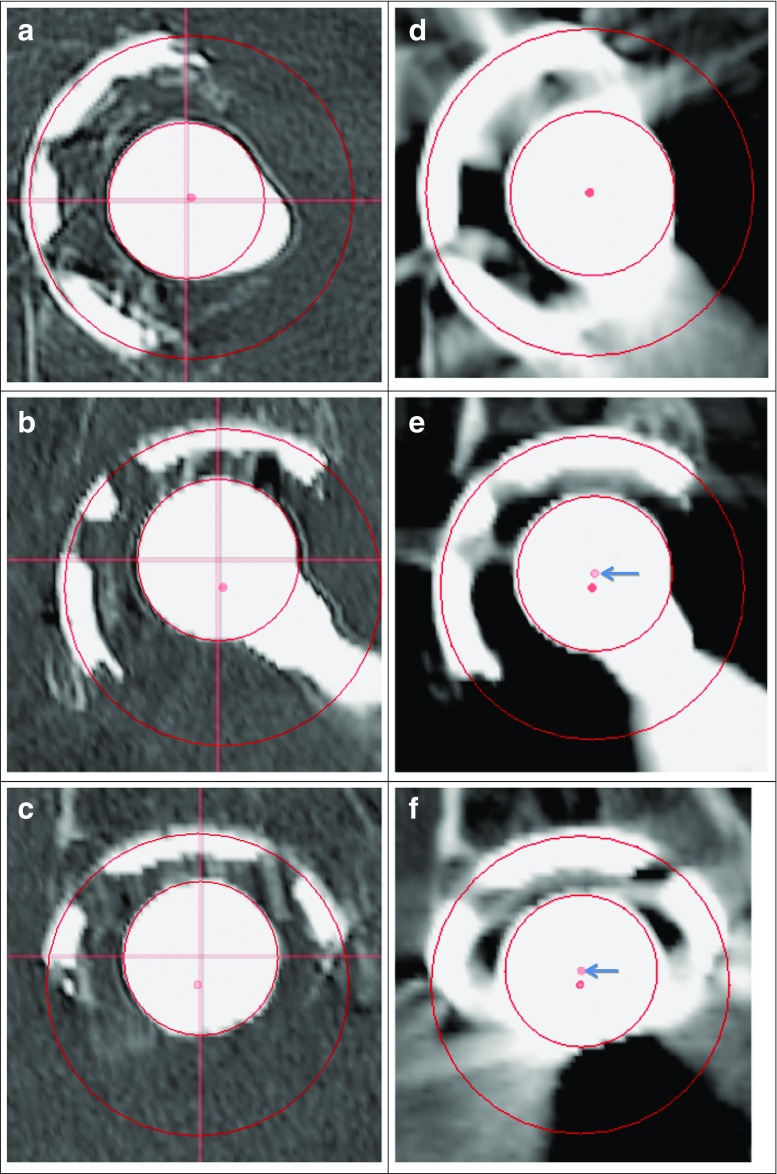
Three orthogonal projections (axial, coronal, and sagittal) with **a**, **b**, and **c** showing the scan from 2017, and **d**, **e**, and **f** showing the scan from 2004. In each of the figures, the *dot* marks the center of the cup. In figure parts **a**, **b**, and **c**, *lines* represent planes through the center of the femoral head. These lines are the projection of the orthogonal planes into the respective image plane. In the plane with the center of the femoral head, all of these lines would intersect. In **e** and **f**, the *dot* (with an *arrow* pointing to it) represents the center of the femoral head. The *circle* around the femoral head represents the projection of a 3D sphere surrounding the femoral head into the respective plane. The *circle around the cup* represents the projection into the respective plane of a 3D sphere, which has the cup as a part of the surface

In preparation for the operation, we performed a final CT examination in March 2017. We analyzed the data as follows. All the CT volumes were rotated so that the pelvis was neutrally oriented based upon the McKibbin plane, and then registered [9] to the 2004 scan. Using the methods described in [10], we located the center of the cup (as part of a sphere) and the center of the femoral head. Assuming that the center of the femoral head and the center of the cup were identical when the prosthesis was implanted, we could directly compute the vector between these two centers. The length of this vector corresponds to the distance the femoral head had penetrated into the cup in each specific year (see Table 1). The X, Y, and Z components of this vector are also shown in Table 1 (X increases from patient left to right, Y increases from back to front, and Z increases from cranial to caudal). In 2004, we can see in Fig. 1e and f that the wear has been in the cranial (Z) direction. The center of the cup and the center of the femoral head in the axial direction are two slices apart; hence they cannot both be shown in the same axial slice. As the slice thickness in 2004 was 1.25 (mm) (see Table 2), this agrees well with the 2.61 mm increase in penetration. The X and Y components of less than 0.2 mm are effectively zero due to the limited accuracy (± 0.05 mm) in the finding of the centers; hence, the magnitude of the difference between two such values can be up to 0.2 mm.^1^ Given the pixel size of 0.68 mm for this scan, this difference is less than one pixel, hence we can see both the femoral head and cup centers in the coronal and sagittal views (arrows in Fig. 1e and f, respectively). In 2017, we can see that there is additional wear in the dorsal (Y) direction and in the lateral (X) direction (see Fig. 1a, b, and c). As part of a protocol to check for prosthetic loosening, the scan from 2017 was performed with the prosthesis bearing leg turned maximally outward to a position that does not cause the patient pain. Because the liner was loose in the cup, part of the increase in the X component may be due to the wear in the liner in the cranial direction rotating into the X direction. However, as we do not image the liner, we cannot be sure of the liner’s actual position, only that the femoral head moved farther in the X direction. The femoral head and the cup centers are one coronal slice apart, two sagittal slices apart, and six axial slices apart; given the 0.36-mm pixel size and 0.5-mm slice thickness (see row for 2017 in Table 2), this corresponds to differences of 0.36, 0.72, and 3.0 mm and is in agreement with the 0.36 mm difference in the Y components, the 1.03 mm difference in the X components, and the 2.29 mm difference in the Z components between 2004 and 2017. To emphasize the distance between the head and cup in the 2017 scan, we have added intersecting lines. These lines are the projection of the orthogonal planes passing through the center of the femoral head onto the respective image plane. In the plane with the center of the femoral head, all of these lines would intersect. One can now easily see the displacement of the center of the femoral head with respect to the center of the cup, even though both centers do not fall into the same planes. Furthermore, simply comparing the distance (of 2.38 mm, Table 1) between the centers of the femoral heads in 2004 and 2017 does not capture the three dimensional direction of the wear. Additionally, the metal artifacts due to the prosthesis are not nearly as prominent in 2017 (Fig. 1a–c) as in 2004 (Fig. 1d–f).

**Table 1:** Summary of wear data. The coordinate system is based on the 2004 scan placed into the standard position of the pelvis and all subsequent scans were aligned to this

Year	X component (mm)	Y component (mm)	Z component (mm)	Additional penetration distance (mm)	Head to cup difference relative to 2004 head–cup difference (mm)
2004	0.20	−0.20	−2.59	2.61	0.00
2005	0.29	0.01	−2.98	2.99	0.39
2007	0.09	−0.30	−3.14	3.15	0.54
2011	−0.13	−0.33	−3.89	3.91	1.30
2016	−0.32	−0.34	−4.45	4.48	1.87
2017	−0.83	−0.56	−4.88	4.99	2.38

The head-to-cup distance is the Euclidean distance between the two points. i.e., the distance between the two three-dimensional points

**Table 2:** CT parameters

CT date	CT manufacturer	CT model (all multislice configuration)	Number of slices	kVp	mAs	Pitch	Revolution time (s)	Slice thickness (mm)	Pixel size (mm)
20041008	General Electric Medical Systems	LightSpeed QX/i	4	120	65.5	0.75	0.800	1.25	0.68
20050926	General Electric Medical Systems	LightSpeed QX/i	4	120	163.8	1.5	0.800	1.25	0.68
20071011	General Electric Medical Systems	LightSpeed VTC	64	120	69.6	0.98	0.600	1.25	0.82
20110113	General Electric Medical Systems	LightSpeed VTC	64	140	48.0	0.98	0.400	0.50	0.78
20160504	Philips Medical Systems	Ingenuity CT	128	120	123.0	0.8	0.500	3.00	0.81
20170310	Toshiba Medical Systems	Aquilion ONE	320	135	37.0	0.5	0.275	0.50	0.36

We planned to change only the polyethylene liner by inserting a new cemented polyethylene cup into the old metal shell. We also planned to replace the modular prosthetic femoral head. In March 2017, her hip prosthesis was revised (by one of the authors) using the same posterior approach as on her initial operation 25 years before. The cup and stem were solidly fixed, but the liner was loose and worn while the prosthetic femoral head was intact. Wear of the retrieved liner was 5.5 mm measured with a micrometer (Cocraft micrometer, 0–25 mm with an accuracy of ±0.04 mm, Cocraft is a brand of Clas Ohlson, Insjön, Sweden) compared to 5 mm measured on the CT examination of the hip 13 days before surgery. The direction of penetration of the prosthetic femoral head into the polyethylene liner changed over time from cranial to dorsal direction. The direction of the measured thinnest part of the retrieved liner coincided with the direction found on the last CT examination. There were no signs of infection and perioperative cultures were negative. The cyst was found and drained but not removed. We cemented a 43-mm polyethylene cup into the acetabular metal shell and changed the prosthetic femoral head to a new 28-mm plus 10 femoral head. The operation took 45 min and the patient was discharged 4 days after surgery. On follow-up examination after 6 weeks she was doing fine overall, but was a little swollen in her left leg and had slight pain.

## Discussion

In our case, repeated CT examinations (using the parameters given in Table 2) provided information that allowed the revision surgery to be limited to liner replacement with almost no loss of blood, as opposed to replacement of the entire prosthesis. This enabled the patient to return to normal life quickly (4 days from operation to release from the hospital). As seen in our data analysis, the direction of wear changed from cranial to dorsal.

An alternative method might be to use magnetic resonance imaging (MRI). However, even with the metal artifact reduction system (MARS) feature, MRI is mostly used for soft tissue diagnosis predominately with metal on metal prostheses. Measurements of liner wear would have been very difficult using MRI. Moreover, CT has better geometric properties, hence is more suited to this application [11, 12].

As planar X-ray has many disadvantages regarding positioning and magnification, we suggest that CT, which demands no special positioning and therefore can be done expeditiously, is preferable. Modern CT scans are quite fast to perform, once the patient is positioned on the scanning couch. The advantages using CT are that much more information about the implant and bone stock is available than with conventional radiographic examination. The disadvantages have *previously* been higher examination costs and higher radiation levels. With a modern CT examination, the level of radiation can be lowered to about the same level as a conventional radiographic examination and the costs for CT examinations are coming down. As an example, the effective dose for the CT scan in 2007 was 7.80 mSv, that for 2011 was 3.46 mSv, and for 2017 was 1.33 mSv. The disadvantages with conventional radiographic examination of the hip are that it takes a longer time to examine the patient, especially if it is necessary to get comparable examinations for the assessment of wear. A modern CT examination of the hip is much faster since the patient position is not crucial. In digital radiographic examinations, each examination is associated with a unique magnification. This makes comparison between different examinations hard, unless each examination is calibrated by including an object with a known size in the picture. In our institution, radiologists prefer CT as it is faster and more reliable. In contrast, positioning of the patient for a planar X-ray can be a problem. The same idea of following the patient with CT would also apply to total knee replacements.

In conclusion, uncemented hip replacements have been used in younger patients for over 20 years. Compared to cemented implants, some of these uncemented implants have been associated with a substantially higher revision rate due to osteolysis and implant loosening. With CT, it is possible to detect osteolysis and wear earlier than with conventional radiographic examination before loosening occurs. It is then possible to monitor the implant condition over time. If a physician wants to know if there is loosening of the bone around a particular patient’s implant and/or if there is wear of the cup, a CT examination should be performed instead of a conventional X-ray of the hip. If wear or bone loss around the implant is detected, we have the opportunity to follow the patient with serial CT examinations.

## Footnotes

1 As there are two pairs of difference measurements.

## References

[1] Puolakka TJ, Pajamäki KJ, Halonen PJ, Pulkkinen PO, Paavolainen P, Nevalainen JK (2001). The Finnish arthroplasty register: report of the hip register. Acta Orthop Scand.

[2] Malchau H, Potter HG. Implant wear symposium 2007 clinical work group. How are wear-related problems diagnosed and what forms of surveillance are necessary? J Am Acad Orthop Surg. 2008;16 Suppl 1:S14–9. 10.5435/00124635-200800001-00005 18612008

[3] Sandgren B, Crafoord J, Olivecrona H, Garellick G, Weidenhielm L. Risk factors for periacetabular osteolysis and wear in asymptomatic patients with uncemented total hip arthroplasties. Scientific World Journal. 2014;2014:905818. 10.1155/2014/905818 PMC4248425 25478600

[4] Clarke JC, Black K, Rennie C, Amstutz HC. Can wear in total hip arthroplasties be assessed from radiographs? Clin Orthop. 1976;126–42. 991493

[5] Jedenmalm A, Noz ME, Olivecrona H, Olivecrona L, Stark A (2008). A new approach for assessment of wear in metal-backed acetabular cups using computed tomography: a phantom study with retrievals. Acta Orthop.

[6] Sandgren B, Skorpil M, Nowik P, Olivecrona H, Crafoord J, Weidenhielm L (2016). Assessment of wear and periacetabular osteolysis using dual energy computed tomography on a pig cadaver to identify the lowest acceptable radiation dose. Bone Joint Res.

[7] Yamaguchi M, Bauer TW, Hashimoto Y (1997). Three-dimensional analysis of multiple wear vectors in retrieved acetabular cups. J Bone Joint Surg Am.

[8] Rahman L, Cobb J, Muirhead-Allwood S (2012). Radiographic methods of wear analysis in total hip arthroplasty. J Am Acad Orthop Surg.

[9] Olivecrona H, Weidenhielm L, Olivecrona L, Beckman MO, Stark A, Noz ME (2004). A new CT method for measuring cup orientation after total hip arthroplasty: a study of 10 patients. Acta Orthop Scand.

[10] Maguire GQ, Noz ME, Olivecrona H, Zeleznik MP, Weidenhielm L (2014). A new automated way to measure polyethylene wear in THA using a high resolution CT scanner: method and analysis. Sci World J.

[11] Goldvasser D, Hansen VJ, Noz ME, Maguire GQ, Zeleznik MP, Olivecrona H (2014). In vivo and ex vivo measurement of polyethylene wear in total hip arthroplasty: comparison of measurements using a CT algorithm, a coordinate-measuring machine, and a micrometer. Acta Orthop.

[12] Goldvasser D, Noz ME, Maguire GQ, Olivecrona H, Bradgon C, Malchau H (2012). A new technique for measuring wear in total hip arthroplasty using computerized tomography. J Arthopasty.

